# Urine Protein-to-Creatinine Ratio Remains Informative Despite Nonsteady State Serum Creatinine During Acute Kidney Injury

**DOI:** 10.1016/j.ekir.2024.09.011

**Published:** 2024-09-21

**Authors:** Ian E. McCoy, Aris Oates, Chi-yuan Hsu

**Affiliations:** 1 Division of Nephrology, University of California San Francisco, San Francisco, California, USA; 2 Division of Pediatric Nephrology, University of California San Francisco, San Francisco, California, USA

**Keywords:** acute kidney injury, nephrotic syndrome, protein-to-creatinine ratio, proteinuria

## Abstract

**Introduction:**

Experts have cautioned that assessment of proteinuria using urine protein-to-creatinine ratios (UPCRs) are not valid during acute kidney injury (AKI) because reduced urine creatinine in the denominator may artificially inflate the ratio. However, there is little empiric data assessing this theoretical concern.

**Methods:**

Here, we retrospectively examined changes in UPCRs measured during episodes of severe AKI and assessed whether the magnitude and direction of these changes associate with how the serum creatinine level is changing at the time of UPCR collection. We repeated these analyses comparing hospitalization UPCRs with prehospitalization or posthospitalization UPCRs, where available.

**Results:**

Among 329 adults hospitalized with stage 2 or 3 AKI (defined as peak:nadir serum creatinine during hospitalization ≥ 2) at the University of California, San Francisco from January 1, 2014 to December 31, 2022 with multiple UPCRs measured during AKI hospitalization, UPCR values were similar whether the serum creatinine was increasing or decreasing at the time of measurement (median difference, 0.06 g/g; interquartile range [IQR], −0.26 to 0.50 g/g). There was no association between the difference in serum creatinine slopes when the UPCRs were collected and the difference in UPCR values (UPCR 0.05 g/g higher per mg/dl/d serum creatinine slope; 95% confidence interval [CI], −0.36 to 0.47, *P* = 0.80). UPCRs measured during hospitalization demonstrated positive and negative predictive values suggesting utility in appraising clinically relevant outpatient UPCR levels.

**Conclusion:**

Despite nonsteady state serum creatinine at the time of collection, UPCRs measured during AKI hospitalizations may be more informative than previously believed and should not be wholly disregarded.

## Graphical abstract

**Figure:**
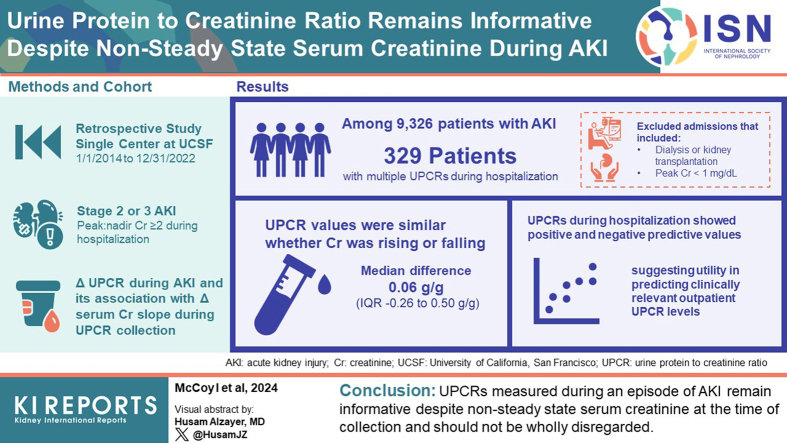

Proteinuria is a key clinical finding that informs both diagnosis^1^ and prognosis^2^^,^^3^ in patients with chronic kidney disease. Measurement of proteinuria during an episode of AKI may be performed for similar reasons. For example, nephrotic-range proteinuria concurrent with AKI may help lead physicians to consider nonsteroidal anti-inflammatory drug-related minimal change disease and acute interstitial nephritis higher up on the differential diagnosis.^4^ On the other hand, falsely concluding that there is a large amount of proteinuria in a patient with AKI may lead to unnecessary invasive work-up such as kidney biopsy.

Furthermore, both preadmission proteinuria^5^ and proteinuria measured 3 months after discharge^6^^,^^7^ have been shown to be prognostically valuable. Despite that, they are often not checked in routine clinical practice. Thus, it would be advantageous to know if proteinuria quantified during an AKI hospitalization could serve as a substitute for prehospitalization or posthospitalization values.

Numerous opinion leaders, however, have cautioned against using UPCR to measure proteinuria during an episode of AKI^8^, ^9^, ^10^ due to concern that the ratio may be artificially affected by acute changes in urine creatinine excretion (Figure 1). The reasoning being that because most protein in the urine is not filtered from the glomerulus and most creatinine in the urine is filtered from the glomerulus, acute decreases in glomerular filtration rate during AKI lead to acutely lower urine creatinine concentration, which would artificially increase the UPCR if urine protein excretion remains unchanged. Alternatively, it is possible that the urine protein in the numerator may decrease in AKI to a similar or even greater manner due to decreased filtered protein load (e.g., in patients pathologically filtering protein at baseline from long-standing diabetic nephropathy) or due to back leak of filtered protein into the peritubular capillaries.^11^ There may be bias in the opposite direction during recovery from AKI: as serum creatinine decreases, higher urine creatinine may lower the UPCR. The extent and variability with which this overestimation or underestimation occurs is not well-described and cannot be determined with simulated data.^10^ Whether UPCRs measured during AKI may represent useful clinical information or mere artifact remains an important, unanswered question.

**Figure 1:**
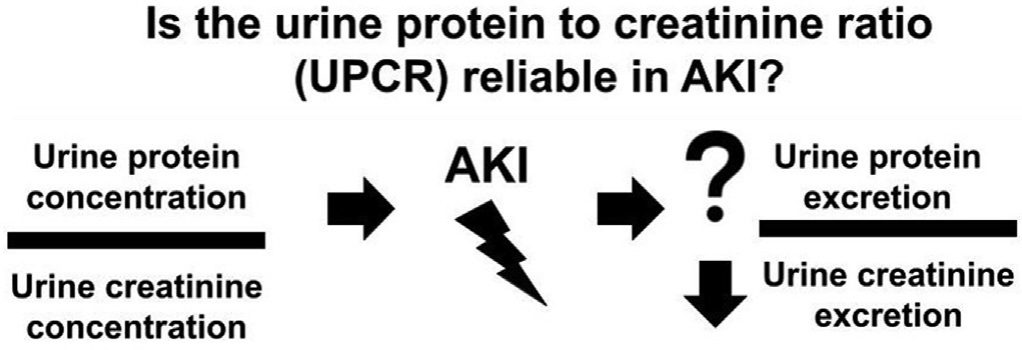
Conceptual changes to UPCR during AKI. AKI is defined by increases in serum creatinine due to decreased urine creatinine excretion, which would artificially increase the UPCR by decreasing the denominator. Less is known about whether proportional changes in urine protein excretion may occur in AKI. AKI, acute kidney injury; UPCR, urine protein-to-creatinine ratio.

To address this gap in the literature, here we examined changes in UPCRs measured during episodes of severe AKI and assessed whether the magnitude and direction of these changes associate with how the serum creatinine level was changing at the time of UPCR collection. We tested the hypothesis that UPCR varies proportionally with the serum creatinine slope at the time of UPCR collection (i.e., the UPCR will be higher when collected during a time period when the serum creatinine is increasing and be lower when the serum creatinine is decreasing). In patients with available outpatient UPCR measurements, we also examined associations between UPCRs measured during AKI hospitalization and UPCRs measured prehospitalization or posthospitalization.

## Methods

### Study Population

We studied clinical information available from the electronic health record on adults (aged ≥ 18 years) hospitalized at the University of California San Francisco between January 1, 2014, and December 31, 2022, with stage 2 or 3 AKI, defined as at least a doubling of the peak relative to the nadir serum creatinine during admission (Supplementary Figure S1). Only 1 hospitalization was analyzed per patient (if a patient had multiple hospitalizations meeting the AKI criteria, then the hospitalization with the most UPCRs was selected). We excluded hospitalizations that included dialysis or kidney transplantation. We also excluded hospitalizations where the peak serum creatinine was < 1 mg/dl after noticing that some patients were misclassified as having AKI due to a single spuriously low nadir value. The study was performed in accordance with the Declaration of Helsinki and was deemed exempt from the requirement for informed consent by the University of California Institutional Review Board (IRB#: 23-38615).

### Comparing UPCRs Measured During Hospitalization

To be included in the study, all patients had to have at least one urine PCR measured during a hospitalization with stage 2 or 3 AKI.

In our primary analysis, we focused on patients with 2 or more UPCRs during hospitalization. For each patient, we selected 2 UPCRs from among all available UPCRs collected as part of routine clinical care during the hospitalization: the one with the steepest upward and the one with the steepest downward serum creatinine slope at the time of UPCR collection. The intent of this design was to maximize the chance of detecting our hypothesized difference in UPCR (i.e., higher UPCR when serum creatinine is increasing and lower UPCR when serum creatinine is decreasing).

The serum creatinine slope was calculated as the change between the closest serum creatinine values before and after the UPCR measurement (requiring at least 6 hours between serum creatinine measurements) divided by the number of hours between the measurements, then multiplied by 24 hours to convert the units from mg/dl/h to the more clinically familiar mg/dl/d.

### Comparing UPCRs Measured During Hospitalization With Outpatient Measurements

We also compared inpatient UPCR with UPCRs measured prehospitalization UPCR (closest to admission date within 1 year prior) and posthospitalization (closest to discharge date within 1 year after) among patients with at least one inpatient and one outpatient UPCR measurement. The inpatient UPCR with the steepest upward serum creatinine slope was used for this analysis since this value would be expected to be the most ‘biased’ by nonsteady state serum creatinine. The serum creatinine slope at the time of outpatient UPCR measurement was assumed to be zero or stable.

### Statistical Analysis

Descriptive statistics were presented as medians (IQR). *P* values for differences were calculated by Wilcoxon signed-rank test for medians and by paired *t* test for means. The associations between within patient differences in UPCRs and the difference in serum creatinine slopes at the time of UPCR collection were assessed using scatterplots with Pearson correlation coefficients and linear regressions (y = mx + b, where y = difference in the 2 UPCRs for each patient, x = difference in serum creatinine slopes at times of UPCR collection for each patient, m = the fitted estimate of the association between y and x, and b = the intercept). No additional covariates were entered in the regression model. In sensitivity analysis, we examined outliers (bottom 5th percentile and top 95th percentile) for the difference between the 2 inpatient UPCR values and for the difference between the inpatient and posthospitalization UPCR values. Test characteristics (sensitivity, specificity, positive predictive value, and negative predictive value) were calculated for clinically relevant inpatient UPCR cutoffs of any proteinuria (UPCR > 0.15 g/g) and nephrotic-range proteinuria (UPCR > 3.0 g/g) to evaluate the associations with the same cutoffs in the outpatient setting. All analyses were performed using SAS/STAT software (Version 9.4 of the SAS System for Windows, 2016).

## Results

Among 9326 patients hospitalized with stage 2 or 3 AKI, UPCR was measured in 19% (*N* =1735; Supplementary Figure S1). Other forms of proteinuria quantification were less common, with UACR measured in 4% of patients and timed (e.g., 24-hour) urine protein measured in 3% of patients. Semi quantitation of urine protein using dipstick urinalysis was common (although it is unclear whether the intent was to quantify proteinuria vs. to detect urinary tract infection or for other purposes) (Supplementary Figure S2).

### Primary Analysis: Comparing Inpatient UPCRs Measured at Different Serum Creatinine Slopes

Among the 329 patients with stage 2 or 3 AKI who had multiple inpatient UPCRs, the median hospital length of stay was 19 days with median peak serum creatinine value of 2.5 mg/dl and median nadir serum creatinine value of 0.8 mg/dl (Table 1). The maximum UPCR measured during hospitalization for each patient was a median of 1.20 g/g (IQR, 0.57–3.03 g/g).

**Table 1:** Characteristics of patients with AKI stage 2 or 3 and multiple inpatient UPCRs

Characteristics	Overall (*N* = 329)
Age at admission, yr	56 (37–67)
Female	52%
**Prehospitalization information**	
Preadmission serum creatinine, mg/dl	1.1 (0.8–1.6) (*n* = 211)
Preadmission UPCR, g/g	0.44 (0.18–1.64) (*n* = 57)
**Hospitalization information**	
Hospitalization length of stay, d	19 (10–34)
Peak inpatient serum creatinine, mg/dl	2.5 (1.8–3.6)
Nadir inpatient serum creatinine, mg/dl	0.8 (0.6–1.1)
Days between peak and nadir creatinine measurements	9 (4–15)
Maximum UPCR, g/g	1.20 (0.57–3.03)
**Posthospitalization information**	
Postdischarge serum creatinine, mg/dl	1.2 (0.8–1.7) (*n* = 209)
Postdischarge UPCR, g/g	0.32 (0.18–1.80) (*n* = 74)

UPCR, urine protein-to-creatinine ratio.

Continuous variables given in median (IQR). Prehospitalization or posthospitalization laboratory results were the closest available within 1 year.

We selected for analysis the UPCR with the steepest upward serum creatinine slope at the time of collection and the UPCR with the steepest downward serum creatinine slope at the time of collection. The median number of hours between creatinine measurements before and after UPCR measurement that were used to calculate the serum creatinine slope was 15 (IQR, 11–24). For the former, median slope was an increase of 0.15 mg/dl per day; for the latter, median slope was a decrease of 0.20 mg/dl per day. The median difference between the 2 UPCR values was 0.06 (IQR, −0.26 to 0.50) g/g (Table 2). The 5th to 95th percentile differences were −2.01 to 4.15 g/g. The 2 UPCR values were strongly correlated (*r* = 0.81; Supplementary Figure S3).

**Table 2:** Pairs of UPCRs (with the steepest upward and steepest downward serum creatinine slope at the time of measurement) among patients with AKI stage 2 or 3 and multiple inpatient UPCRs (*n* = 329)

Characteristics	UPCR with the steepest upward serum creatinine slope at the time of measurement	UPCR with the steepest downward serum creatinine slope at the time of measurement	*P* value
Days between the 2 UPCR measurements, median (IQR)	3.20 (1.28–8.67)		
Serum creatinine slope at time of UPCR collection, mg/dl/d, median (IQR)	0.15 (0.00–0.35)	−0.20 (−0.53 to −0.04)	
Slope difference, mg/dl/d, median (IQR)	0.37 (0.16–0.83)		<0.0001
UPCR, g/g, median (IQR)	0.91 (0.37–2.28)	0.74 (0.34–1.95)	
UPCR Difference, g/g, median (IQR)	0.06 (−0.26 to 0.50)		0.01
Difference in UPCR by difference in serum creatinine slope (g/g per mg/dl/d), estimate (95% confidence interval)	0.05 (−0.36 to 0.47)		0.80
In subset analysis among those with slope difference > 1 mg/dl/d (*n* = 66), estimate (95% confidence interval)	0.21 (−0.24 to 0.66)		0.94

IQR, interquartile range; UPCR, urine protein-to-creatinine ratio.

Results shown as observed median (IQR: 25th percentile–75th percentile) or estimated beta-coefficient (95% confidence interval) from linear regression.

**Figure 2:**
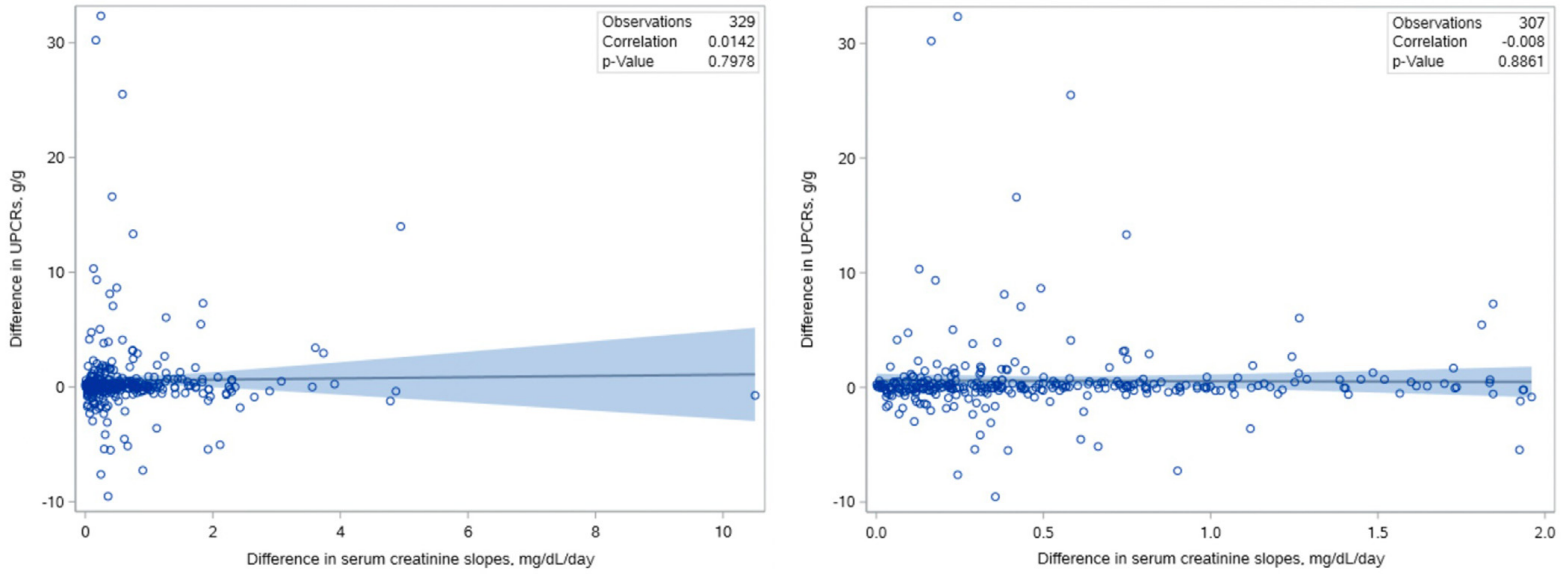
Scatter plot of the difference in serum creatinine slopes at the times of UPCR measurement versus the difference in UPCR values (left panel) and excluding 22 outliers with a difference in serum creatinine slopes > 2 mg/dl/d (right panel). No association between the difference in serum creatinine slopes at the times of UPCR measurement (steepest upward slope − steepest downward slope, in mg/dl/d) and difference in UPCRs (UPCR with steepest upward slope − UPCR with steepest downward slope, in g/g). Regression line shown with 95% confidence interval. UPCR, urine protein-to-creatinine ratio.

A linear regression found no significant association between the difference in serum creatinine slopes when the UPCRs were collected and the difference in UPCR values (Figure 2 and Table 2; UPCR 0.05 g/g higher per mg/dl/d steeper upward serum creatinine slope; 95% CI, −0.36 to 0.47, *P* = 0.80). Even in the subgroup with more extreme differences in serum creatinine slopes (> 1 mg/dl/d), results were similar: 0.21 g/g higher UPCR per mg/dl/d of serum creatinine slope difference; 95% CI, −0.24 to 0.66, *P* = 0.94.

### Secondary Analysis: Comparing Inpatient UPCRs With Outpatient UPCRs

**Figure 3:**
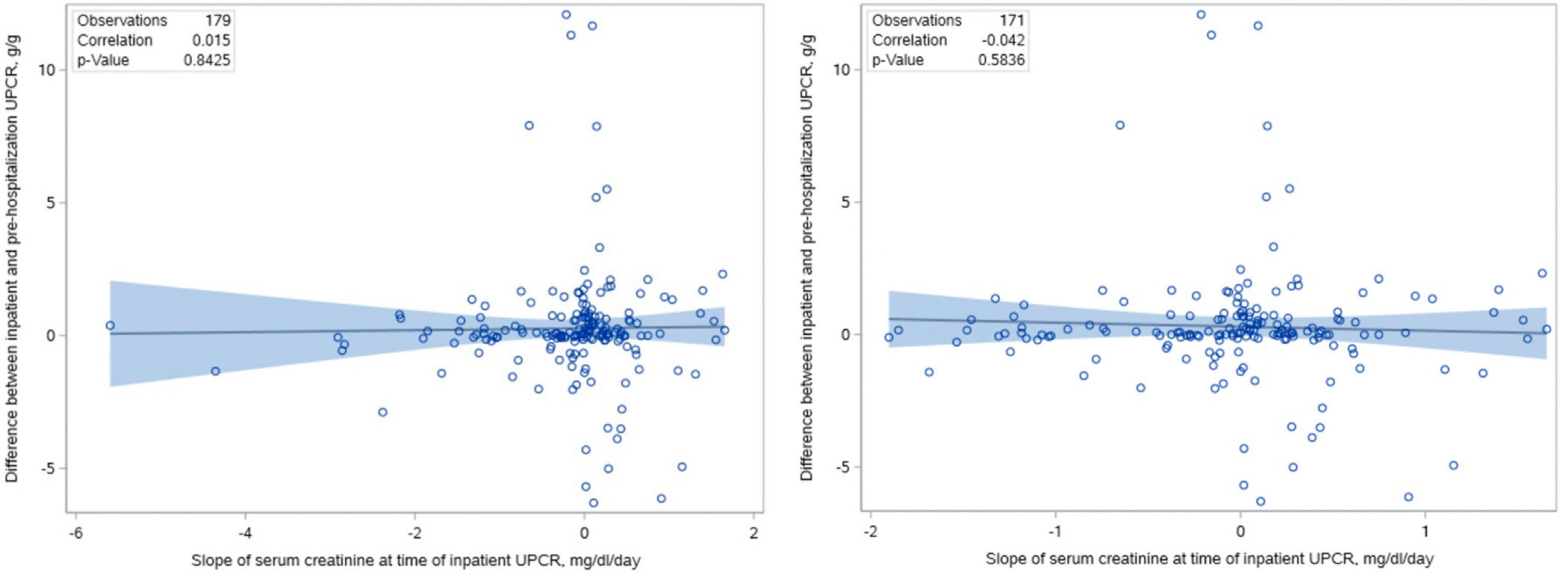
Scatter plot of the serum creatinine slope at the time of inpatient UPCR measurement versus the difference between the inpatient and prehospitalization UPCR (left panel) and excluding 8 outliers with a serum creatinine slope < −2 mg/dl/d (right panel). No association between the serum creatinine slope at the time of inpatient UPCR measurement (in mg/dl/d) and the difference between the inpatient and prehospitalization UPCR (inpatient UPCR − posthospitalization UPCR, in g/g). Regression line shown with 95% confidence interval. UPCR, urine protein-to-creatinine ratio.

Among 179 patients with at least one inpatient UPCR and one prehospitalization UPCR (Supplementary Figure S4) measured a median of 72 (IQR, 18–173) days before admission, inpatient UPCRs were slightly higher than prehospitalization UPCRs (Table 1 and Table 3) with a median difference of 0.07 g/g. However, similar to the analysis of inpatient UPCRs (Table 2), the magnitude of the difference did not depend on the serum creatinine slope at the time of inpatient UPCR collection (Figure 3; 0.04 g/g higher per mg/dl/d; 95% CI, −0.33 to 0.40, *P* = 0.84). An inpatient UPCR with an abnormal amount of proteinuria (UPCR > 0.15 g/g) associated with a prehospitalization UPCR > 0.15 g/g with 94% sensitivity, 27% specificity, 83% positive predictive value, and 53% negative predictive value (Supplementary Table S1). An inpatient UPCR with nephrotic-range proteinuria (UPCR > 3.0 g/g) associated with prehospitalization UPCR > 3.0 g/g with 45% sensitivity, 96% specificity, 59% positive predictive value, and 93% negative predictive value (Supplementary Table S2).

**Table 3:** UPCRs measured during hospitalization versus prehospitalization in patients with AKI stage 2 or 3 (*n* = 179)

Characteristics	Inpatient UPCR with the steepest upward serum creatinine slope at the time of measurement	Prehospitalization UPCR	*P* value
UPCR, g/g, median (IQR)	0.72 (0.26–1.54)	0.33 (0.18–1.17)	
UPCR difference (inpatient − outpatient), g/g, median (IQR)	0.07 (−0.16 to 0.70)		0.01
UPCR difference in inpatient UPCR relative to outpatient UPCR per mg/dl/d higher inpatient serum creatinine slope (g/g per mg/dl/d), estimate (95% confidence interval)	0.04 (−0.33 to 0.40)		0.84

IQR, interquartile range; UPCR, urine protein-to-creatinine ratio.

Results shown as observed median (IQR: 25th percentile–75th percentile) or estimated beta-coefficient (95% confidence interval) from linear regression.

**Figure 4:**
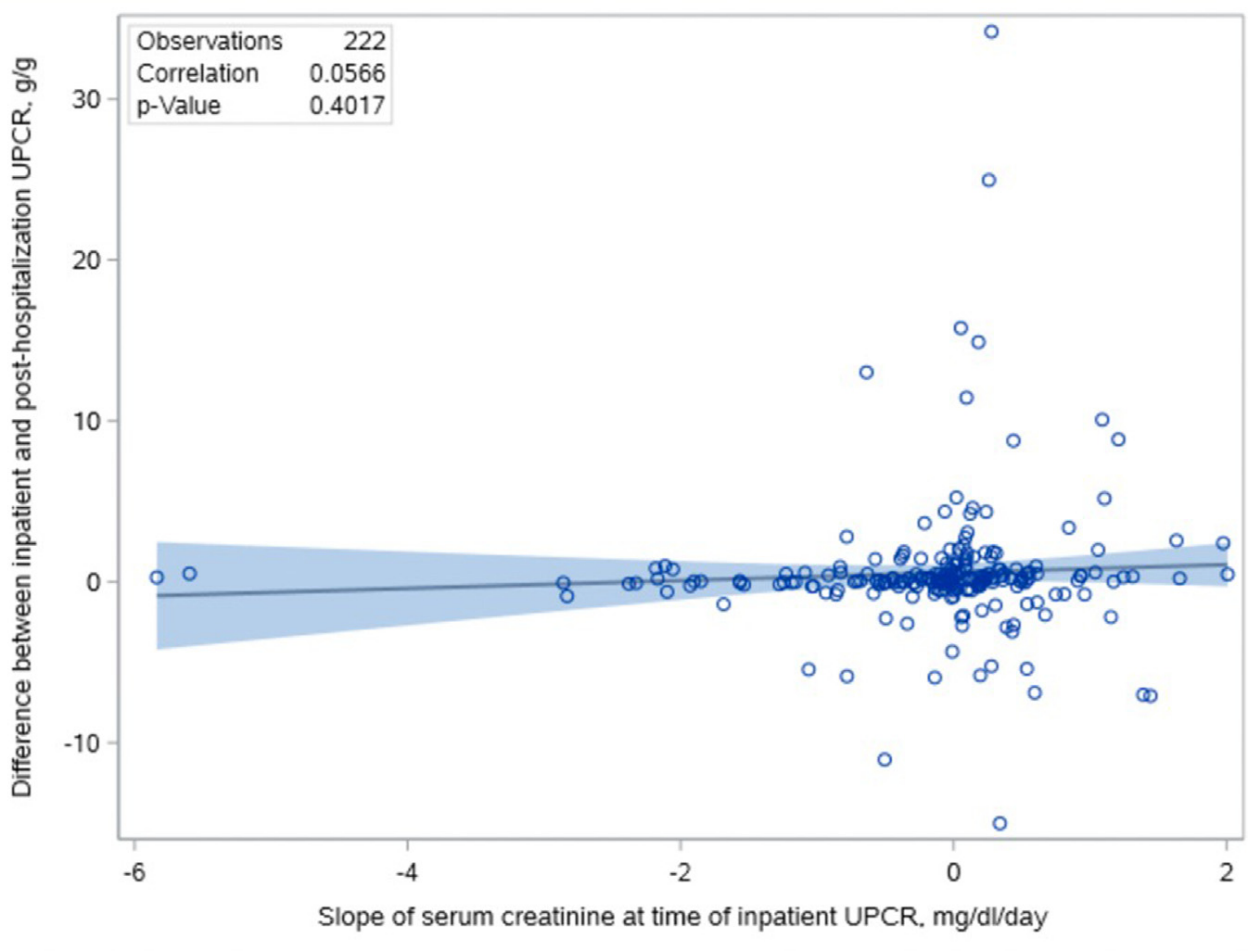
Scatter plot of the serum creatinine slope at the time of inpatient UPCR measurement versus the difference between the inpatient and posthospitalization UPCR. No association between the serum creatinine slope at the time of inpatient UPCR measurement (in mg/dl/d) and the difference between the inpatient and posthospitalization UPCR (inpatient UPCR − posthospitalization UPCR, in g/g). Regression line shown with 95% confidence interval. UPCR, urine protein-to-creatinine ratio.

Among 222 with at least one inpatient UPCR and one posthospitalization UPCR (Supplementary Figure S4) measured a median of 46 (IQR, 14–130) days after discharge, inpatient UPCRs were slightly higher than posthospitalization UPCRs (Table 1 and Table 4) with a median difference of 0.10 g/g. However, similar to the analysis of inpatient UPCRs (Table 2), the magnitude of the difference did not depend on the serum creatinine slope at the time of inpatient UPCR collection (Figure 4; 0.25 g/g higher per mg/dl/d; 95% CI, −0.33 to 0.83, *P* = 0.40). An inpatient UPCR with an abnormal amount of proteinuria (UPCR > 0.15 g/g) associated with a posthospitalization UPCR > 0.15 g/g with 93% sensitivity, 16% specificity, 79% positive predictive value, and 60% negative predictive value (Supplementary Table S3). An inpatient UPCR with nephrotic-range proteinuria (UPCR > 3.0 g/g) associated with posthospitalization UPCR > 3.0 g/g with 47% sensitivity, 91% specificity, 45% positive predictive value, and 92% negative predictive value (Supplementary Table S4).

**Table 4:** UPCRs measured during hospitalization versus posthospitalization in patients with AKI stage 2 or 3 (*n* = 222)

Characteristics	Inpatient UPCR with the steepest upward serum creatinine slope at the time of measurement	Posthospitalization UPCR	*P* value
UPCR, g/g, median (IQR)	0.65 (0.28–1.73)	0.33 (0.17–1.22)	
UPCR difference (inpatient − outpatient), g/g, median (IQR)	0.10 (−0.24 to 0.63)		0.004
UPCR difference in inpatient UPCR relative to outpatient UPCR per mg/dl/d higher inpatient serum creatinine slope (g/g per mg/dl/d), estimate (95% confidence interval)	0.25 (−0.33 to 0.83)		0.40

IQR, interquartile range; UPCR, urine protein-to-creatinine ratio.

Results shown as observed median (IQR: 25th percentile–75th percentile) or estimated beta-coefficient (95% confidence interval) from linear regression.

### Outlier Analyses

We also analyzed 33 outlier patients, the bottom 5th and top 95th percentile differences between the 2 inpatient UPCRs (Supplementary Table S5). Compared to the entire cohort, the outliers had similar serum creatinine slopes at the times of UPCR collection. Although they had large differences between the 2 inpatient UPCRs (by definition, because this difference was used to define them as outliers) with a median difference 4.15 g/g, most did not have a normal UPCR (minimum UPCR during hospitalization was > 0.15 g/g for 94% of the outlier patients, > 1.00 g/g for 79%, > 3.00 g/g for 55%); thus, it was not as if 1 UPCR was “spuriously” high and the recheck UPCR was normal. Eleven of these outlier patients had timed urine protein collections during hospitalization, with a median of 4.6 g/24 h and a minimum and maximum of 1.4 g/24 h and 10.0 g/24 h, respectively.

When we repeated the outliers for the “inpatient UPCR minus posthospitalization UPCR” difference (23 patients above the 95th [> 4.59 g/g] or below the 5th percentile [< −4.34 g/g]), the posthospitalization UPCR was still > 0.15 g/g in 96%, > 1.00 g/g in 83%, and > 3.00 g/g in 70%.

## Discussion

Despite the theoretical limitations of UPCR during an AKI episode, we found that UPCRs are being ordered by clinicians in real-life clinical practice. Contrary to conventional wisdom, we found no association between the difference in UPCR measurements and the difference in the rate of change of serum creatinine at the times of UPCR collection (despite a median difference in serum creatinine slope of about 0.4 mg/dl/d). Results were consistent whether an inpatient UPCR was being compared with another inpatient UPCR or with an outpatient UPCR value and in subgroup analyses looking at the most extreme serum creatinine slopes. Among outlier patients with the largest differences between inpatient UPCRs, we found that the minimum UPCR during hospitalization was still nephrotic in 55% of patients and abnormal in 94% of patients, demonstrating that markedly elevated UPCR measuring during AKI remain informative despite nonsteady state serum creatinine.

Our findings suggest that the transient decrease in urinary creatinine excretion known to occur in AKI may be matched by a proportional decrease in urinary protein excretion, as has been suggested by others,^11^^,^^12^ thereby keeping the UPCR relatively stable. Potential explanations include a decreased filtered protein load (in patients pathologically filtering protein at baseline such as those with long-standing diabetic nephropathy) and backleak of filtered protein into the peritubular capillaries. For patients with multiple inpatient UPCRs, the difference between the values measured with the steepest upward and steepest downward serum creatinine slopes was a median of only 0.06 g/g. Patients with rapidly changing serum creatinine levels did not have larger differences in inpatient UPCRs than patients with relatively more stable serum creatinine levels had. Thus, the theoretical fear that UPCR systematically overestimates true proteinuria when the serum creatinine is increasing and systematically underestimates it when the serum creatinine is decreasing seems to be overstated.

Although we observed large differences between multiple inpatient UPCRs in a minority of cases (∼3 g/g in the 10% with the largest differences), these larger differences were not more common in patients with larger differences in serum creatinine slope at the times of UPCR measurement. Whether these larger differences between UPCRs measured in the same patient are due to true changes in renal pathology (e.g., tubular proteinuria from AKI) remains unclear; however, they do not seem to be related to the nonsteady state serum creatinine at the time of UPCR measurement. Several pieces of evidence suggest that they are also not due to spurious measurement errors. For example, among the patients with large differences in inpatient UPCRs due to 1 of the UPCRs being nephrotic-range, most (55%) had nephrotic-range proteinuria on all the UPCRs checked during hospitalization. Furthermore, in the subset whose physicians ordered a timed urine protein collection, the median protein excretion in 24 hours was of nephrotic range. Most patients (70%) with large differences between inpatient and posthospitalization UPCRs also had nephrotic-range proteinuria on the posthospitalization measurement. Thus, it appears that UPCRs may be more variable at higher or nephrotic-range values (similar to what has been observed for outpatient UACR values^13^^,^^14^); however, this variability is not related to the stability of the serum creatinine at the time of UPCR collection. Clinically, this variability may be less important because UPCR results of 3 g/g versus 6 g/g are unlikely to affect clinical decision-making. Based on these data, it would seem unwarranted to discount UPCR results measured during AKI entirely.

Given that UPCR use is established in the outpatient setting, we compared the correlation among UPCRs measured during AKI hospitalizations to the correlation among UPCRs measured in the outpatient setting. Despite being measured when the serum creatinine was increasing versus decreasing with a median difference in serum creatinine slopes of about 0.4 mg/dl/d, the 2 inpatient UPCRs showed strong correlation (*r* = 0.81, Supplementary Figure S3). This degree of correlation was higher than what we observed between prehospitalization and posthospitalization outpatient values (*r* = 0.65, Supplementary Figure S5). For comparison, correlation coefficients between spot UPCRs and 24-hour urine protein collections vary from 0.56 to 0.98;^15^ nevertheless, UPCR has been accepted as having equivalent or better clinical utility as a 24-hour urine protein excretion.^15^ Although urine creatinine excretion is obviously transiently decreased as AKI develops and transiently increased as AKI recovers, urine creatinine excretion also varies in the absence of AKI^10^ and may change hour by hour due to variance in diet, volume status, and other factors.^16^ These factors contribute to the increasingly recognized large within-person variability of UACRs in stable outpatients.^14^^,^^17^ Thus, UPCRs measured during AKI likely retain clinical utility.

Although in an ideal world all patients with AKI would have UPCRs measured in clinic in the prehospitalization setting to better predict AKI recovery and in the posthospitalization setting to inform risk prognostication as recommended by Kidney Disease: Improving Global Outcomes guidelines,^18^ in practice this rarely happens.^19^ Consequently, providers caring for patients with AKI in the hospital are often left with only inpatient UPCRs. We found that an inpatient UPCR measured in the setting of stage 2 or 3 AKI ruled out outpatient nephrotic-range proteinuria more than 90% of the time if the inpatient value was < 3.0 g/g. An inpatient UPCR measured in the setting of stage 2 or 3 AKI > 0.15 g/g associated with an abnormally elevated outpatient proteinuria level 79% of the time. These results suggest that inpatient UPCR measurements may be useful surrogates for outpatient UPCR values, though future multicenter studies measuring UPCRs in unselected patients with AKI (rather than this retrospective analysis of patients in whom clinicians chose to measure multiple UPCRs) are needed to confirm or refute these findings.

It is important to note several limitations of our study. First, we did not have sequentially timed urine protein and creatinine excretion measurements, so we cannot report how serum creatinine slope associates with changes in urine protein and urine creatinine excretion separately. Second, AKI may have a true effect on the UPCR value apart from any measurement error introduced by the decreased urine creatinine excretion, such as tubular proteinuria seen with tubular necrosis. However, one would expect this to bias toward finding an association between serum creatinine slope and UPCR, which we did not observe. Third, it is possible that urine was made on one day and collected on another (i.e., sat in the bladder for an extended period) especially in patients with oliguric AKI without foley catheters, which could contribute to dissociation between UPCR and the serum creatinine slope on the day of collection. Fourth, it is likely that we have ascertainment bias in these retrospectively analyzed UPCR measurements (i.e., UPCR measurements were not ordered in a standardized way such as according to a research protocol, but rather physicians chose to measure UPCR recurrently in certain patients and not in others as part of routine clinical care). However, this effect likely biases toward seeing a greater discrepancy between UPCR measurements since clinicians were probably more likely to recheck a UPCR if a first UPCR value seemed unusually high. Thus, our results likely overestimate the discrepancy one might observe between repeated UPCR values in an unselected population (i.e., it is unlikely that this type of bias resulted in underestimation of UPCR discrepancy here). Our results should not be extrapolated to urine analytes besides protein because the concentrations of other urinary biomarkers, which are often also indexed to the urine creatinine concentration, may change in different ways during AKI.^10^ Finally, our analyses used data from a single academic center and should be replicated in larger, multicenter data.

In conclusion, we were unable to detect any association between UPCR and the serum creatinine slope at the time of UPCR collection in patients with AKI. Although many of us can recall a patient with a surprisingly high UPCR measured during AKI that normalized on recheck, based on our analysis, these spurious results seem to be relatively rare and unrelated to the serum creatinine slope at the time of UPCR collection. UPCRs measured during AKI hospitalizations may be more informative than previously believed and should not be wholly disregarded.

## Disclosure

All the authors declared no competing interests.

## Supplementary Material

Supplementary File (PDF) **Figure S1.** Cohort assembly. **Figure S2.** Measures used to assess proteinuria in stage 2 or 3 AKI. **Figure S3.** Scatter plot of the inpatient UPCR measured with the steepest upward serum creatinine slope and the inpatient UPCR measured with the steepest downward serum creatinine slope for the 329 patients with at least 2 inpatient UPCRs (left panel) and excluding those with UPCRs ≥ 10 g/g (right panel). **Figure S4.** Venn diagram of the 3 overlapping cohorts used for the primary and secondary analyses. **Figure S5.** Scatter plot of the prehospitalization UPCR versus the posthospitalization outpatient UPCR for the 99 patients with at least 1 prehospitalization and 1 posthospitalization UPCR. **Table S1.** 2 × 2 table showing test characteristics of inpatient UPCR > 0.15 g/g for prehospitalization UPCR > 0.15 g/g (*n* = 179). **Table S2.** 2 × 2 table showing test characteristics of inpatient UPCR > 3.0 g/g for prehospitalization UPCR > 3.0 g/g (*n* = 179). **Table S3.** 2 × 2 table showing test characteristics of inpatient UPCR > 0.15 g/g for posthospitalization UPCR > 0.15 g/g (*n* = 222). **Table S4.** 2 × 2 table showing test characteristics of inpatient UPCR > 3.0 g/g for posthospitalization UPCR > 3.0 g/g (*n* = 222). **Table S5.** Characteristics of outliers (patients with AKI stage 2 or 3 and at least 2 inpatient UPCRs whose differences in the UPCRs collected with maximum or minimum serum creatinine slopes were above the 95th [>4.15 g/g] or below the 5th percentile [< −2.01 g/g]). **STROBE Checklist.**

## Footnotes

Supplementary File (PDF)
**Figure S1.** Cohort assembly. **Figure S2.** Measures used to assess proteinuria in stage 2 or 3 AKI. **Figure S3.** Scatter plot of the inpatient UPCR measured with the steepest upward serum creatinine slope and the inpatient UPCR measured with the steepest downward serum creatinine slope for the 329 patients with at least 2 inpatient UPCRs (left panel) and excluding those with UPCRs ≥ 10 g/g (right panel). **Figure S4.** Venn diagram of the 3 overlapping cohorts used for the primary and secondary analyses. **Figure S5.** Scatter plot of the prehospitalization UPCR versus the posthospitalization outpatient UPCR for the 99 patients with at least 1 prehospitalization and 1 posthospitalization UPCR. **Table S1**. 2 × 2 table showing test characteristics of inpatient UPCR > 0.15 g/g for prehospitalization UPCR > 0.15 g/g (*n* = 179). **Table S2.** 2 × 2 table showing test characteristics of inpatient UPCR > 3.0 g/g for prehospitalization UPCR > 3.0 g/g (*n* = 179). **Table S3**. 2 × 2 table showing test characteristics of inpatient UPCR > 0.15 g/g for posthospitalization UPCR > 0.15 g/g (*n* = 222). **Table S4.** 2 × 2 table showing test characteristics of inpatient UPCR > 3.0 g/g for posthospitalization UPCR > 3.0 g/g (*n* = 222). **Table S5.** Characteristics of outliers (patients with AKI stage 2 or 3 and at least 2 inpatient UPCRs whose differences in the UPCRs collected with maximum or minimum serum creatinine slopes were above the 95th [>4.15 g/g] or below the 5th percentile [< −2.01 g/g]).
**STROBE Checklist.**
