# Supplementary material for: Urine Protein-to-Creatinine Ratio Remains Informative Despite Nonsteady State Serum Creatinine During Acute Kidney Injury
Source: Kidney Int Rep. 2024 Sep 21;9(12):3455–63. doi: 10.1016/j.ekir.2024.09.011 (PMC11652066; doi:10.1016/j.ekir.2024.09.011)
Supplement: Supplementary File (PDF) — Figure S1. Cohort assembly. Figure S2. Measures used to assess proteinuria in stage 2 or 3 AKI. Figure S3. Scatter plot of the inpatient UPCR measured with the steepest upward serum creatinine slope and the inpatient UPCR measured with the steepest downward serum creatinine slope for the 329 patients with at least 2 inpatient UPCRs (left panel) and excluding those with UPCRs ≥ 10 g/g (right panel). Figure S4. Venn diagram of the 3 overlapping cohorts used for the primary and secondary analyses. Figure S5. Scatter plot of the prehospitalization UPCR versus the posthospitalization outpatient UPCR for the 99 patients with at least 1 prehospitalization and 1 posthospitalization UPCR. Table S1. 2 × 2 table showing test characteristics of inpatient UPCR > 0.15 g/g for prehospitalization UPCR > 0.15 g/g (n = 179). Table S2. 2 × 2 table showing test characteristics of inpatient UPCR > 3.0 g/g for prehospitalization UPCR > 3.0 g/g (n = 179). Table S3. 2 × 2 table showing test characteristics of inpatient UPCR > 0.15 g/g for posthospitalization UPCR > 0.15 g/g (n = 222). Table S4. 2 × 2 table showing test characteristics of inpatient UPCR > 3.0 g/g for posthospitalization UPCR > 3.0 g/g (n = 222). Table S5. Characteristics of outliers (patients with AKI stage 2 or 3 and at least 2 inpatient UPCRs whose differences in the UPCRs collected with maximum or minimum serum creatinine slopes were above the 95th [>4.15 g/g] or below the 5th percentile [< −2.01 g/g]). STROBE Checklist. [file mmc1.pdf]

Supplemental Material

Figure S1. Cohort assembly

Figure S2. Measures used to assess proteinuria in stage 2/3 AKI.

Figure S3. Scatter plot of the inpatient UPCR measured with the steepest upward serum creatinine slope and the inpatient UPCR measured with the steepest downward serum creatinine slope for the 329 patients with at least two inpatient UPCRs (left panel) and excluding those with UPCRs ≥ 10 g/g (right panel).

Figure S4. Venn diagram of the 3 overlapping cohorts used for the primary and secondary analyses.

Figure S5. Scatter plot of the pre-hospitalization UPCR versus the post-hospitalization outpatient UPCR for the 99 patients with at least one pre-hospitalization and one post-hospitalization UPCR.

Table S1. 2x2 Table showing test characteristics of Inpatient UPCR > 0.15 g/g to predict pre-hospitalization UPCR > 0.15 g/g (n=179)

Table S2. 2x2 Table showing test characteristics of Inpatient UPCR > 3.0 g/g to predict pre-hospitalization UPCR > 3.0 g/g (n=179)

Table S3. 2x2 Table showing test characteristics of Inpatient UPCR > 0.15 g/g to predict post-hospitalization UPCR > 0.15 g/g (n=222)

Table S4. 2x2 Table showing test characteristics of Inpatient UPCR > 3.0 g/g to predict post-hospitalization UPCR > 3.0 g/g (n=222)

Table S5. Characteristics of Outliers (patients with AKI stage 2/3 and at least two inpatient UPCRs whose difference in the UPCRs collected with maximum/minimum serum creatinine slopes was above the 95th (>4.15 g/g) or below the 5th percentile (<-2.01 g/g)

STROBE Checklist.

Figure S1. Cohort assembly

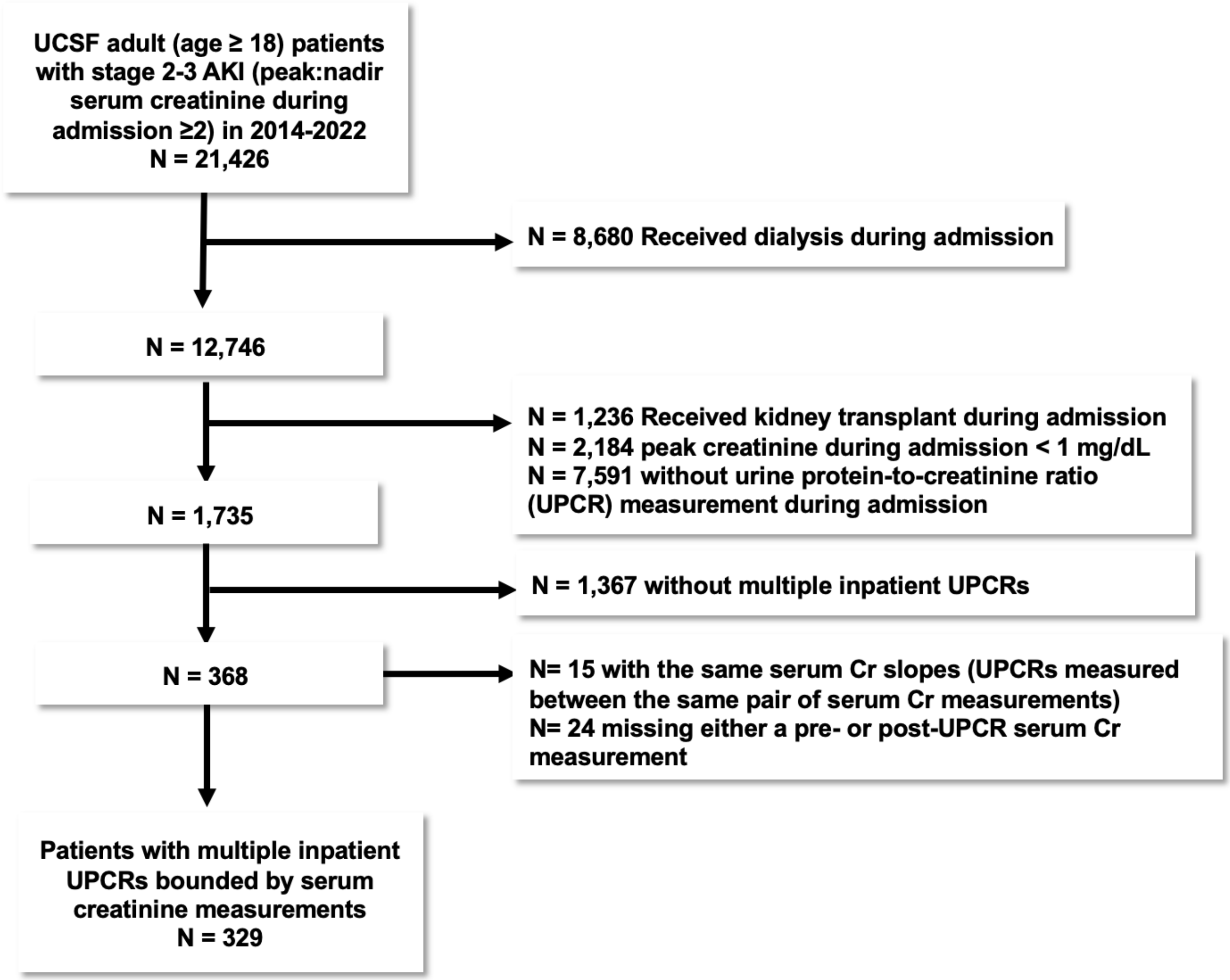

Figure S2. Measures used to assess proteinuria in stage 2/3 AKI

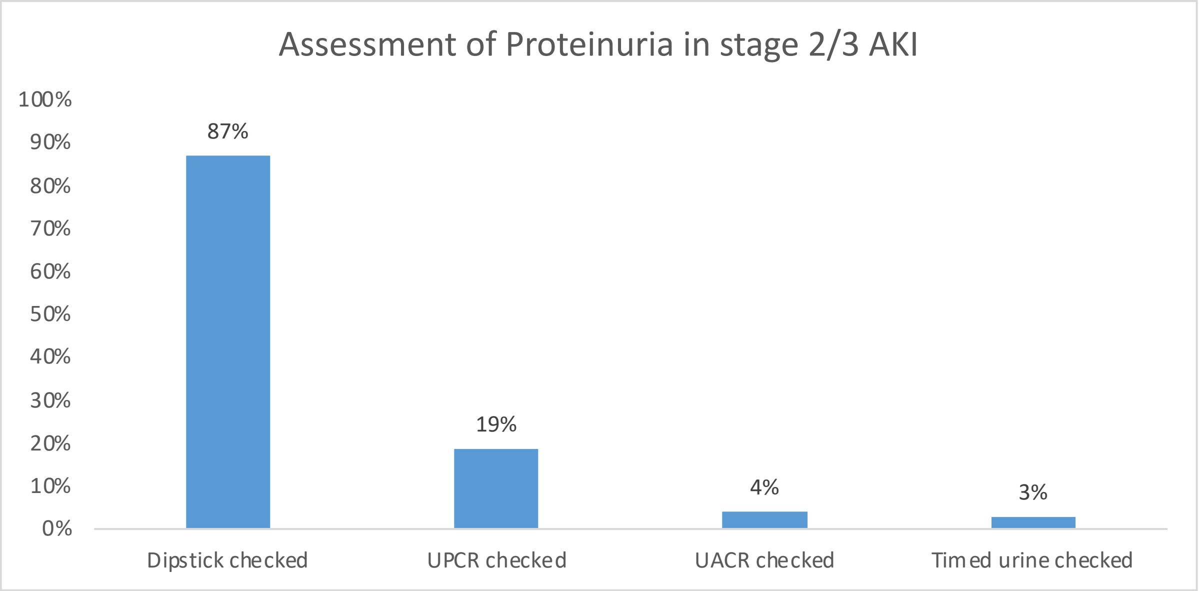

N=9,326 adults with stage 2/3 AKI who did not have dialysis or transplant during hospitalization and had a peak serum creatinine >= 1 mg/dL. UPCR: urine protein to creatinine ratio. UACR: urine albumin to creatinine ratio.

Figure S3. Scatter plot of the inpatient UPCR measured with the steepest upward serum creatinine slope and the inpatient UPCR measured with the steepest downward serum creatinine slope for the 329 patients with at least two inpatient UPCRs (left panel) and excluding those with UPCRs ≥ 10 g/g (right panel).

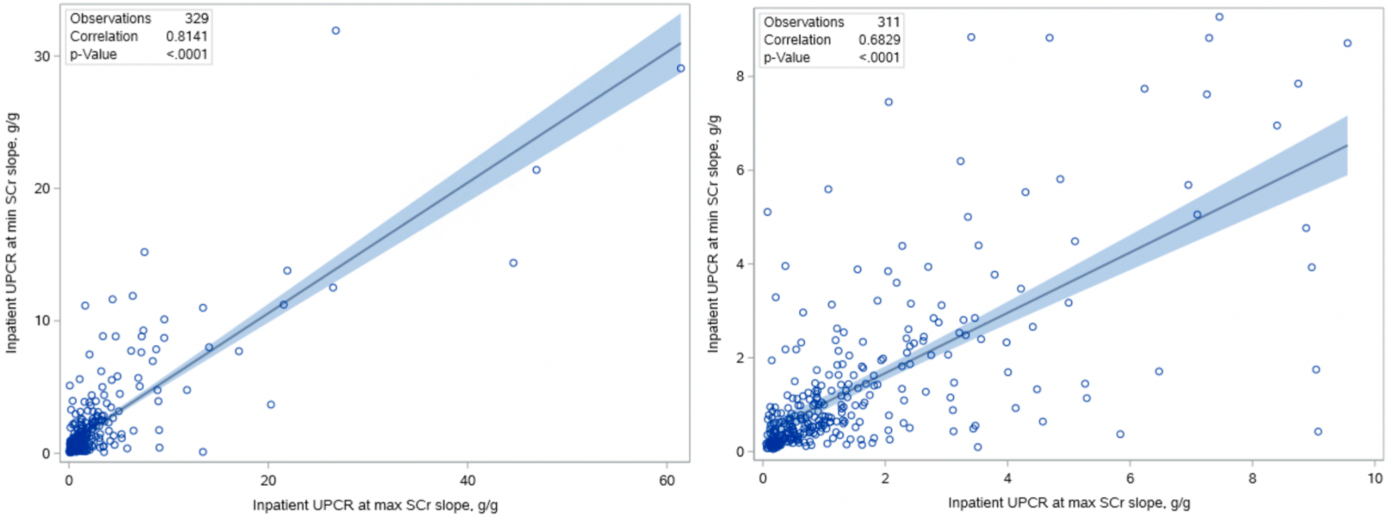

Regression lines shown with 95% confidence interval.

Figure S4. Venn diagram of the 3 overlapping cohorts used for the primary and secondary analyses.

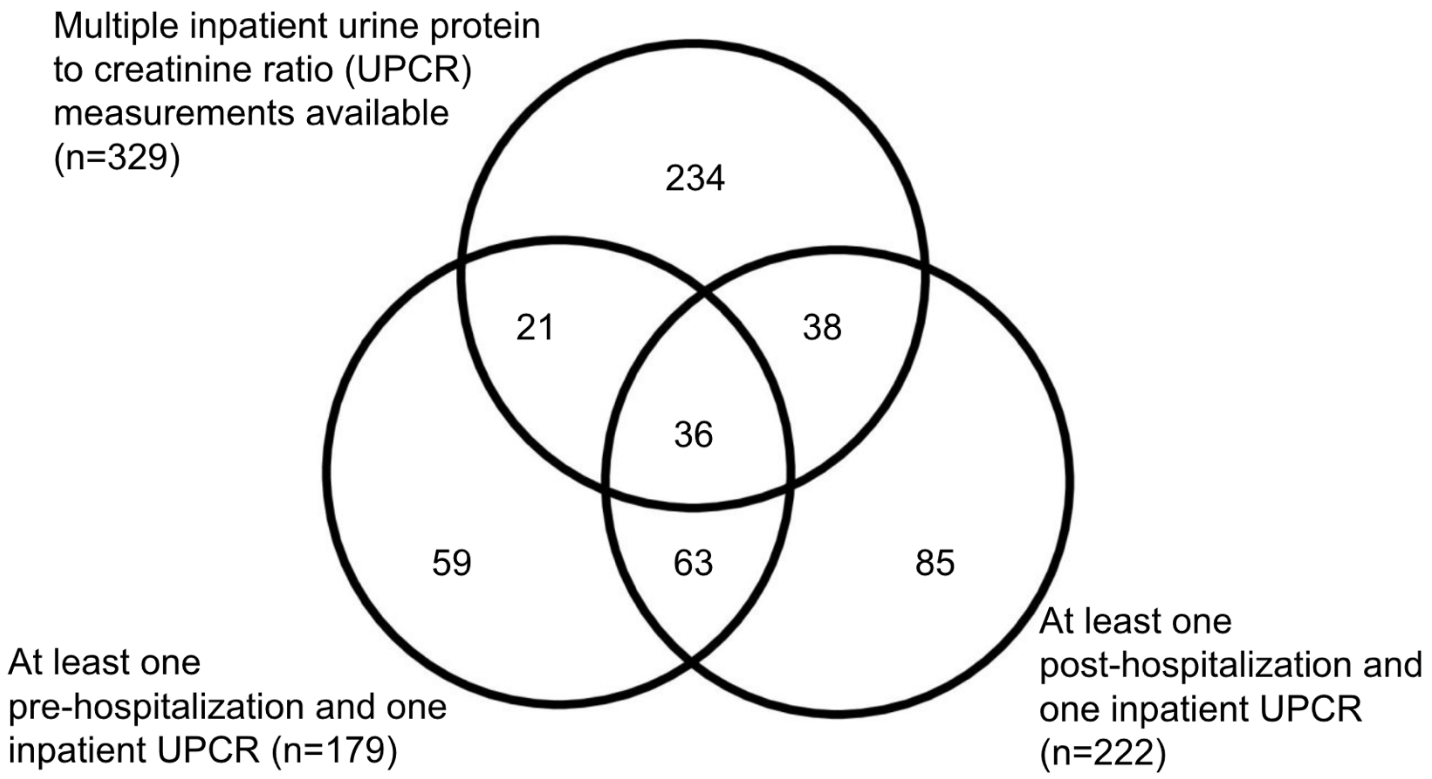

Figure S5. Scatter plot of the pre-hospitalization UPCR versus the post-hospitalization outpatient UPCR for the 99 patients with at least one pre-hospitalization and one post-hospitalization UPCR.

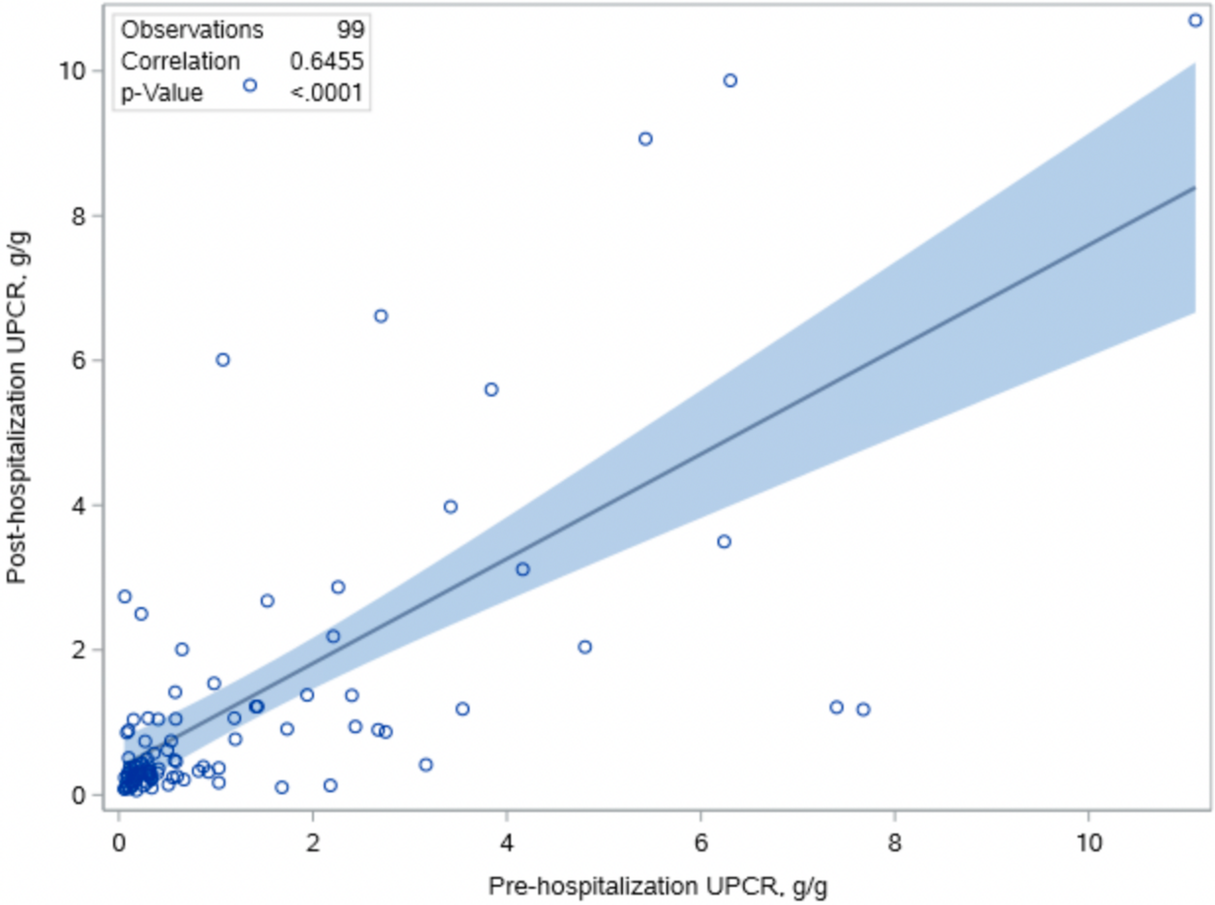

Regression line shown with 95% confidence interval.

Table S1. 2x2 Table showing test characteristics of Inpatient UPCR > 0.15 g/g to predict pre-hospitalization UPCR > 0.15 g/g (n=179)

		**Pre-hospitalization UPCR > 0.15 g/g**		**Predictive values**
		Yes	No	
**Inpatient UPCR > 0.15 g/g**	Yes	133	27	PPV=133/(133+27) = 83%
	No	9	10	NPV = 10/(10+9) = 53%
**Sensitivity and Specificity**		Sn = 133/(133+9) =94%	Sp=10/(27+10) =27%	

When more than one inpatient UPCR was available, the measurement collected at maximum serum creatinine slope was used. Sn: sensitivity; Sp: specificity; PPV: positive predictive value; NPV: negative predictive value.

Table S2. 2x2 Table showing test characteristics of Inpatient UPCR > 3.0 g/g to predict pre-hospitalization UPCR > 3.0 g/g (n=179)

		**Pre-hospitalization UPCR > 3.0 g/g**		**Predictive values**
		Yes	No	
**Inpatient UPCR > 3.0 g/g**	Yes	10	7	PPV=10/(10+7) = 59%
	No	12	150	NPV = 150/(12+150) = 93%
**Sensitivity and Specificity**		Sn = 10/(10+12) =45%	Sp=150/(7+150) =96%	

When more than one inpatient UPCR was available, the measurement collected at maximum serum creatinine slope was used. Sn: sensitivity; Sp: specificity; PPV: positive predictive value; NPV: negative predictive value.

Table S3. 2x2 Table showing test characteristics of Inpatient UPCR > 0.15 g/g to predict post-hospitalization UPCR > 0.15 g/g (n=222)

		**Post-hospitalization UPCR > 0.15 g/g**		**Predictive values**
		Yes	No	
**Inpatient UPCR > 0.15 g/g**	Yes	159	43	PPV=159/(159+43) = 79%
	No	12	8	NPV = 12/(12+8) = 60%
**Sensitivity and Specificity**		Sn = 159/(159+12) =93%	Sp=8/(43+8) =16%	

When more than one inpatient UPCR was available, the measurement collected at maximum serum creatinine slope was used. Sn: sensitivity; Sp: specificity; PPV: positive predictive value; NPV: negative predictive value.

Table S4. 2x2 Table showing test characteristics of Inpatient UPCR > 3.0 g/g to predict post-hospitalization UPCR > 3.0 g/g (n=222)

		Post-hospitalization UPCR > 3.0 g/g		Predictive values
		Yes	No	
**Inpatient UPCR > 3.0 g/g**	Yes	14	17	PPV=14/(14+17) = 45%
	No	16	175	NPV = 175/(16+175) = 92%
**Sensitivity and Specificity**		Sn = 14/(14+16) =47%	Sp=175/(17+ 175) =91%	

When more than one inpatient UPCR was available, the measurement collected at maximum serum creatinine slope was used. Sn: sensitivity; Sp: specificity; PPV: positive predictive value; NPV: negative predictive value.

Table S5. Characteristics of Outliers (patients with AKI stage 2/3 and at least two inpatient UPCRs whose difference in the UPCRs collected with maximum/minimum serum creatinine slopes was above the 95th (>4.15 g/g) or below the 5th percentile (<-2.01 g/g)

Characteristics	Outliers for inpatient UPCR difference (N = 33)	All (N=329)
**Age at admission, yrs**	45 [32-60]	56 [37-67]
**Female**	61%	52%
**<u>Pre-hospitalization information</u>**		
**Pre-admission serum creatinine, mg/dL**	1.4 [0.7-1.8] (n=18)	1.1 [0.8-1.6] (n=211)
**Pre-admission UPCR, g/g**	0.17 [0.17-0.17] (n=1)	0.44 [0.18-1.64] (n=57)
**<u>Hospitalization information</u>**		
**Hospitalization length of stay, days**	23 [9-37]	19 [10-34]
**Peak inpatient serum creatinine, mg/dL**	3.4 [2.1-3.9]	2.5 [1.8-3.6]
**Nadir inpatient serum creatinine, mg/dL**	0.9 [0.6-1.4]	0.8 [0.6-1.1]
**Maximum UPCR, g/g**	11.15 [5.84-21.49]	1.20 [0.57-3.03]
**Minimum UPCR, g/g**	3.40 [1.14-7.70]	0.52 [0.21-1.33]
**Difference in UPCRs collected with max/min serum creatinine slopes, g/g**	4.15 [-4.53 to 8.65]	0.06 [-0.26 to 0.50]
**Serum Cr slope difference, mg/dL/day**	0.39 [0.24-0.75]	0.37 [0.16 to 0.83]
**<u>Post-hospitalization information</u>**		
**Post-admission serum creatinine, mg/dL**	1.5 [1.1-1.7] (n=21)	1.2 [0.8-1.7] (n=209)
**Post-admission UPCR, g/g**	1.54 [0.31-6.83] (n=12)	0.32 [0.18-1.80] (n=74)

Continuous variables given in median [IQR]. Pre/post admission laboratory results were the closest available within 1 year. UPCR: urine protein to creatinine ratio.

Reporting checklist for cross sectional study.

Based on the STROBE cross sectional guidelines.

Instructions to authors

Complete this checklist by entering the page numbers from your manuscript where readers will find each of the items listed below.

Your article may not currently address all the items on the checklist. Please modify your text to include the missing information. If you are certain that an item does not apply, please write "n/a" and provide a short explanation.

Upload your completed checklist as an extra file when you submit to a journal.

In your methods section, say that you used the STROBE cross sectionalreporting guidelines, and cite them as:

von Elm E, Altman DG, Egger M, Pocock SJ, Gotzsche PC, Vandenbroucke JP. The Strengthening the Reporting of Observational Studies in Epidemiology (STROBE) Statement: guidelines for reporting observational studies.

		Reporting Item	Page Number
**Title and abstract**			
Title	#1a	Indicate the study's design with a commonly used term in the title or the abstract	2
Abstract	#1b	Provide in the abstract an informative and balanced summary of what was done and what was found	2
**Introduction**			
Background / rationale	#2	Explain the scientific background and rationale for the investigation being reported	4-5
Objectives	#3	State specific objectives, including any prespecified hypotheses	5
**Methods**			

Study design	#4	Present key elements of study design early in the paper	5-7
Setting	#5	Describe the setting, locations, and relevant dates, including periods of recruitment, exposure, follow-up, and data collection	6
Eligibility criteria	#6a	Give the eligibility criteria, and the sources and methods of selection of participants.	6
	#7	Clearly define all outcomes, exposures, predictors, potential confounders, and effect modifiers. Give diagnostic criteria, if applicable	6-7
Data sources / measurement	#8	For each variable of interest give sources of data and details of methods of assessment (measurement). Describe comparability of assessment methods if there is more than one group. Give information separately for for exposed and unexposed groups if applicable.	6
Bias	#9	Describe any efforts to address potential sources of bias	6-7
Study size	#10	Explain how the study size was arrived at	6,9
Quantitative variables	#11	Explain how quantitative variables were handled in the analyses. If applicable, describe which groupings were chosen, and why	6-8
Statistical methods	#12a	Describe all statistical methods, including those used to control for confounding	7-8
Statistical methods	#12b	Describe any methods used to examine subgroups and interactions	7
Statistical methods	#12c	Explain how missing data were addressed	6
Statistical methods	#12d	If applicable, describe analytical methods taking account of sampling strategy	n/a
Statistical methods	#12e	Describe any sensitivity analyses	7

Results

Participants	#13a	Report numbers of individuals at each stage of study—eg numbers potentially eligible, examined for eligibility, confirmed eligible, included in the study, completing follow-up, and analysed. Give information separately for for exposed and unexposed groups if applicable.	9-12
Participants	#13b	Give reasons for non-participation at each stage	9-12
Participants	#13c	Consider use of a flow diagram	27
Descriptive data	#14a	Give characteristics of study participants (eg demographic, clinical, social) and information on exposures and potential confounders. Give information separately for exposed and unexposed groups if applicable.	9,20
Descriptive data	#14b	Indicate number of participants with missing data for each variable of interest	20, 27
Outcome data	#15	Report numbers of outcome events or summary measures. Give information separately for exposed and unexposed groups if applicable.	9-12
Main results	#16a	Give unadjusted estimates and, if applicable, confounder-adjusted estimates and their precision (eg, 95% confidence interval). Make clear which confounders were adjusted for and why they were included	9-12
Main results	#16b	Report category boundaries when continuous variables were categorized	10-12
Main results	#16c	If relevant, consider translating estimates of relative risk into absolute risk for a meaningful time period	n/a
Other analyses	#17	Report other analyses done—e.g., analyses of subgroups and interactions, and sensitivity analyses	10-12
**Discussion**			
Key results	#18	Summarise key results with reference to study objectives	13
Limitations	#19	Discuss limitations of the study, taking into account sources of potential bias or imprecision. Discuss both direction and magnitude of any potential bias.	16-17

Interpretation	#20	Give a cautious overall interpretation considering objectives, limitations, multiplicity of analyses, results from similar studies, and other relevant evidence.	13-17
Generalisability	#21	Discuss the generalisability (external validity) of the study results	16-17
**Other Information**			
Funding	#22	Give the source of funding and the role of the funders for the present study and, if applicable, for the original study on which the present article is based	18

None The STROBE checklist is distributed under the terms of the Creative Commons Attribution License CC-BY. This checklist can be completed online using https://www.goodreports.org/, a tool made by the EQUATOR Network in collaboration with Penelope.ai
